# Epigenetic and Transcriptomic Characterization of Pure Adipocyte Fractions From Obese Pigs Identifies Candidate Pathways Controlling Metabolism

**DOI:** 10.3389/fgene.2019.01268

**Published:** 2019-12-17

**Authors:** Mette Juul Jacobsen, Jakob H. Havgaard, Christian Anthon, Caroline M. Junker Mentzel, Susanna Cirera, Poula Maltha Krogh, Sachin Pundhir, Peter Karlskov-Mortensen, Camilla S. Bruun, Philippe Lesnik, Maryse Guerin, Jan Gorodkin, Claus B. Jørgensen, Merete Fredholm, Romain Barrès

**Affiliations:** ^1^Department of Veterinary and Animal Sciences, Faculty of Health and Medical Sciences, University of Copenhagen, Copenhagen, Denmark; ^2^Institute of Cardiometabolism and Nutrition (ICAN), Pierre and Marie Curie University, Pitié-Salpetrière Hospital, Paris, France; ^3^Novo Nordisk Foundation Centre for Basic Metabolic Research, Faculty of Health and Medical Sciences, University of Copenhagen, Copenhagen, Denmark

**Keywords:** *Sus scrofa*, DNA methylation, obesity, RNAseq, epigenetics, metabolism

## Abstract

Reprogramming of adipocyte function in obesity is implicated in metabolic disorders like type 2 diabetes. Here, we used the pig, an animal model sharing many physiological and pathophysiological similarities with humans, to perform in-depth epigenomic and transcriptomic characterization of pure adipocyte fractions. Using a combined DNA methylation capture sequencing and Reduced Representation bisulfite sequencing (RRBS) strategy in 11 lean and 12 obese pigs, we identified in 3529 differentially methylated regions (DMRs) located at close proximity to-, or within genes in the adipocytes. By sequencing of the transcriptome from the same fraction of isolated adipocytes, we identified 276 differentially expressed transcripts with at least one or more DMR. These transcripts were over-represented in gene pathways related to MAPK, metabolic and insulin signaling. Using a candidate gene approach, we further characterized 13 genes potentially regulated by DNA methylation and identified putative transcription factor binding sites that could be affected by the differential methylation in obesity. Our data constitute a valuable resource for further investigations aiming to delineate the epigenetic etiology of metabolic disorders.

## Introduction

Obesity is a severe global health concern associated to several metabolic diseases, such as insulin resistance, type 2 diabetes, cardiovascular diseases, and several forms of cancer (Davoodi et al., 2013; Pedersen, 2013). Obesity is defined as a complex disease, and is associated with profound alterations in gene expression, which are caused by both genetic and environmental factors. In humans, genetic factors determine approximately 40% to 70% of the phenotypic variation in obesity (Albuquerque et al., 2015); however, so far, only a small fraction of this variation has been explained by loci identified by genome wide association studies (GWAS). Epigenetic modifications such as DNA methylation and histone modifications have an important influence on gene regulation and have therefore been acknowledged as additional contributing factors in the development of obesity (Campión et al., 2009; Ronn et al., 2015). Hence, genetic variations and DNA methylation may synergistically influence gene regulation and contribute to diseases like obesity and its co-morbidities (Drong et al., 2013; Grundberg et al., 2013; de Toro-Martín et al., 2016; Guénard et al., 2017).

The adipose tissue itself plays an essential role in the development of obesity, not only for the obvious contribution to energy metabolism, but also in the regulation of tissue inflammation and appetite control through the production and secretion of hormones, cytokines, and chemokines (Reilly and Saltiel, 2017). Gene expression in adipose tissue changes significantly in the obese state, due to adipocytes expansion and increased exposure of circulating insulin levels (Ahima and Flier, 2000; Reilly and Saltiel, 2017). These signaling molecules activate different cell surface receptors and drive signaling through the PI3K, MAPK, and JAK/STAT pathways, which are fundamental for cell migration, survival, proliferation, and apoptosis. Thus, long term disturbance of these pathways has considerable consequences for the cells, notably on pathways disrupted in cancer, which may explain why obesity is linked to an increased risk of developing cancer (Hopkins et al., 2016).

However, since adipose tissue is a mixture of several different cell types, including mature adipocytes, preadipocytes, endothelial, and immune cells, it is still unclear how each of the individual cell types contributes to the dysregulation of the pathways associated with obesity. Likewise, the epigenetic profile can also differ substantially between various cell types. Hence profiling DNA methylation in the entire tissue would reflect the overall tissue methylation pattern rather than true differences in methylation in the individual cell types, and due to the large differences in cell composition between lean and obese conditions, it would mask the true cell specific differential methylation.

Therefore, we have made a cell-type specific genome-wide study of mature adipocytes isolated from abdominal fat tissue from obese and lean pigs to explore to which degree the adipocyte expression pattern is perturbed between lean and obese individuals. Differences in methylation pattern were merged with the gene expression level in the very same samples. We show that a large number of genes and pathways are deregulated when comparing expression in adipocytes from lean and obese pigs, respectively. We propose DNA methylation remodeling is a potential factor contributing to gene reprogramming and disease predisposition.

## Materials and Methods

### Animals and Collection of Cells

**Figure 1:**
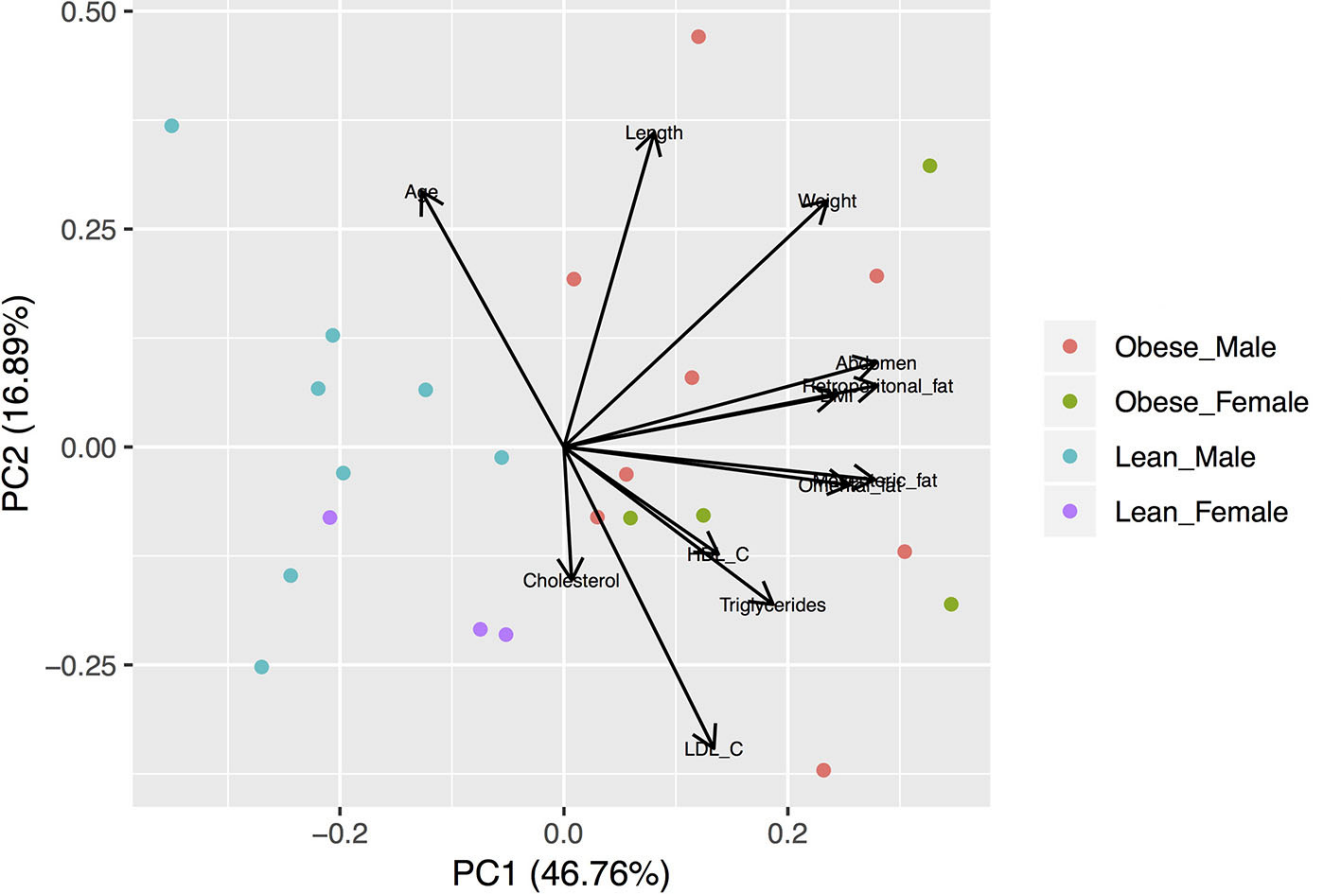
Biplot of the Principal Component Analysis (PCA) of the anthropometric and metabolic characteristics of studied animals. The variables were scaled prior to the analysis. Lean animals are separated from obese animals according to the first component.

24 Duroc-Göttingen minipig inter-cross F2 animals were selected from the UNIK resource population (Kogelman et al., 2012) to represent the two extremes of the lean and obese phenotypes, respectively. There were 8 boars and 4 sows in each group, however one lean female was excluded in the upstream data analysis due to incorrect phenotyping, leaving 12 obese and 11 lean animals for the analysis (**Table 1**, **Figure 1**). The UNIK resource population is a large F2 comprising a total of 502 F2 pigs. It has been generated by crossing the obesity prone Göttingen minipig with lean production pigs (Yorkshire and Duroc) in the parent generation and by intercrossing the F1 animals. All pigs were housed in the same environment and had free access to food and water during their whole lifespan.

**Table 1:** Anthropometric and metabolic characteristic of the animals.

All animals:	Lean (n = 11)	Obese (n = 12)	P value
Age (*months*)	9.88 ± 1.6	9.26 ± 0.6	P = 0.22
Length (*cm*)	87.3 ± 4.5	89.3 ± 8.1	P = 0.48
Weight (*kg*)	84.9 ± 11.7	121 ± 20.7	P = 4.0E−5
BMI	111 ± 12.6	152 ± 10.7	P = 3.6E−6
Abdomen (*cm*)	110 ± 5.8	136 ± 7.5	P = 3.5E−9
Retroperitoneal fat (*g*)	1055 ± 520	3900 ± 1366	P = 2.0E−6
Mesenteric fat (*g*)	12.4 ± 4.3	25.8 ± 8.0	P = 6.5E−5
Omental fat (*g*)	268 ± 139	653 ± 321	P = 1.5E−3
Total Cholesterol (*mg/dl*)	82.6 ± 22.3	104 ± 22.2	P = 0.04
Triglycerides (*mg/dl*)	38.3 ± 8.9	65.7 ± 34.2	P = 0.02
HDL-C (*mg/dl*)	50.0 ± 12.2	55.5 ± 12.0	P = 0.30
LDL-C (*mg/dl*)	24.9 ± 12.6	35.3 ± 12.4	P = 0.06
**RNAseq males**	**Lean (n = 5)**	**Obese (n = 5)**	**P value**
Age month (*months*)	10.48 ± 2.0	9.61 ± 0.50	P = 0.23
Length (*cm*)	85.3 ± 5,2	89.2 ± 5.5	P = 0.30
Weight (*kg*)	77.7 ± 9.9	121.9 ± 11.9	P = 2.1E−4
BMI	106 ± 6.9	153 ± 6.7	P = 4.7E−6
Abdomen (*cm*)	105 ± 4.0	136 ± 6.6	P = 1.6E−5
Retroperitoneal fat (*g*)	718 ± 318	2977 ± 1419	P = 8.4E−3
Mesenteric fat (*g*)	9.5 ± 4.2	22.9 ± 8.3	P = 1.2E−2
Omental fat (*g*)	187 ± 82	585 ± 311	P = 2.4E−2
Total Cholesterol (*mg/dl*)	82.0 ± 19.8	104 ± 18.1	P = 0.10
Triglycerides (*mg/dl*)	32.8 ± 7.6	80.6 ± 38.3	P = 2.6E−2
HDL-C (*mg/dl*)	54.0 ± 13.6	53.6 ± 6.9	P = 0.95
LDL-C (*mg/dl*)	21.4 ± 8.4	34.3 ± 14.0	P = 0.12

The animals were weighted and measured before slaughter, and after slaughter the weight of the visceral fat pads were measured. Triglycerides, total cholesterol, high-density lipoprotein (HDL-C), and low-density lipoprotein (LDL-C) were measured from plasma collected after an overnight fast. Data are mean ± SD. The P-values were calculated as independent t-test.

Slaughtering took place at a commercial slaughterhouse at the ages between 8 and 13 months at which point weight, length, and abdominal circumference were measured. The animals were euthanized by bleeding after electrical stunning, and the following visceral fat samples were collected and weighed: retroperitoneal fat, mesenteric fat, and greater omentum. The latter was measured from an 8-cm diameter large section of the omentum in the triangle between ileum and cecum. All research involving both animal and tissue sampling were conducted according to the Danish “Animal Maintenance Act” (Act 432 dated 09/06/2004) and with the approval from the Danish Animal Experimental Board (J.nr 2007/561-1434).

### Plasma Lipids

Plasma lipid profile was established using an Autoanalyzer (Konelab 20, Thermo Fisher Scientific, Waltham, MA, USA) and commercial kits from Roche Diagnostics for total cholesterol (Roche Diagnostics, Basel, Switzerland) and from Thermo Electron (Thermo Fisher Scientific) for triglycerides (TG) and HDL-cholesterol (HDL-C). Fasting plasma LDL-C was calculated using the Friedewald formula (Friedewald et al., 1972).

### Cell Isolation

Mature adipocytes were isolated from approx. 15 g of the retroperitoneal adipose tissue as described in (Decaunes et al., 2011) with a few adjustments. Briefly, the adipose tissue was quickly removed from the animal during slaughter and rinsed in 37°C PBS (1% penicillin, 1% streptomycin, 1% BSA). The tissue was then roughly minced, and placed in 50 ml falcon tubes, and 20 ml of pre-warmed (37°C) 0.2% collagenase solution (HBSS, 1% BSA) was added. Samples were incubated under shaking for approximately 90 min in a 37°C water bath. The digested adipose tissue was filtered through a 200-µm sterile nylon filter, and washed with DMEM (10% FBS, 4.5 g/L glucose), and the mature adipocytes were allowed to float. These were washed gently three times with DMEM (10% FBS, 4.5 g/L glucose) and allowed time to float to the top after each wash. Adipocyte pellets were hereafter preserved in −80°C until the RNA and DNA extraction. A representable example of the isolated adipocytes is shown in **Supplementary Figure S1**.

### RNA and DNA Extraction

For the global DNA methylation studies, DNA was extracted from the mature adipocytes using the Epicenter kit (Illumina) according to the manufacture's recommendations, except for adding an additional centrifugation step at 4°C after nuclei lysis to remove the released lipids. For the global transcriptome analysis and subsequent quantitative real-time PCR (qPCR) validation, total RNA was isolated from the mature adipocytes according to the protocol described in (Cirera, 2013) including the DNAse treatment step. Nucleic acid concentrations and purity were estimated using the NanoDrop 1000 spectrophotometer (Thermo Scientific) and the RNA quality was further determined using the Experion™ system (Bio-Rad). Only RNA samples with an RQI value above 6 were processed further.

### DNA Methylation Immunoprecipitation Sequencing (mDNAcap)

1.1 µg DNA from each animal was fractionated in a Bioruptor sonicator (Diagenode) into 100- to 500-bp fragments. Methylated DNA fragments were hereafter enriched using the MethylMiner Methylated DNA Enrichment Kit (Invitrogen) according to the protocol. DNA libraries were generated according to the Low Input TruSeq Library Preparation protocol (Illumina) and subjected to single-end (SE) sequencing using Illumina HiSeq2000 and a 100-bp read length. The raw reads were trimmed for quality and adapters using trimmomatic V0.22 (Bolger et al., 2014) (quality trimming by phred quality of 20) The cleaned reads were mapped to the pig genome version 10.2 using segemehl V0.1.4 (Hoffmann et al., 2009) and the following parameters: accuracy 95%, and minimum mapping size 18. The mapped reads were analyzed with diffReps V1.55.4 using the G-test and default values for all remaining parameters (Shen et al., 2013).

### Reduced Representation Bisulfite Sequencing (RRBS)

200-ng genomic DNA from each of the 23 samples was digested with MspI (NEB) and used for gel-free multiplexed reduced representation bisulfite sequencing (mRRBS) as described in (Boyle et al., 2012) with a few modifications. Briefly, the digested products were subjected to end-repair and A-tailing, and adapter–ligated using TruSeq preparations kit (Illumina). Libraries were then pooled and bisulfite treated using EZ DNA methylation Kit for 20 hours (Zymo Research). The converted DNA was amplified using Pfu Turbo Cx (Agilent), purified using a 1× volume of AMPure XP (Beckman Coulter) and subjected to SE sequencing using a HiSeq2000 (Illumina). The raw reads were cleaned for quality and adapters using and in-house tools removing TrueSeq adapters and clipping read tails of phred quality less than 20. The reads were mapped to the pig genome version 10.2 using bismark v 0.10 (Krueger and Andrews, 2011) with standard parameters except using bowtie 2 v2.1.0 for the mapping option of bismark (Langmead and Salzberg, 2012). A minimum of 5 reads were required before calling methylation levels. Mapped reads were analyzed using Metilene v 0.2-7 requiring at least five pigs in both the obese and lean groups (–X 5 –Y 5) (Jühling et al., 2016).

### RNA Sequencing

RNAseq libraries from 10 of the samples (only males, 5 lean and 5 obese) were prepared using the TruSeq stranded total RNA kit including Ribo-Zero Gold (Illumina). Each library was subjected to 100 bp paired-end (PE) sequencing on a HiSeq2000. Six lanes in total were sequenced. The raw reads were cleaned for quality and adapters using and in-house tools removing TrueSeq adapters and clipping read tails of phred quality less than 20 The reads were mapped to the pig transcriptome using the STAR aligner v2.3.1 (Dobin et al., 2013) with the following options and data: pig genome version 10.2, ensembl annotation version 70 and with intron-motifs [XS : A] tags in the SAM output. The aligned reads were assigned to transcripts of the ensembl version 70 annotation with cufflinks version 2.2.0 (Trapnell et al., 2013). The subsequent differential expression analysis was performed with cuffdiff from the cufflinks package and was based on the Ensembl v70 annotation. Standard options were used for the cufflinks programs in all cases. All further analysis was done using the annotation from ensemble v81.

### Pathway Analysis

Webgestalt GSAT analysis 2013 (Zhang et al., 2005) was used to perform functional enrichment analysis of the genes, which were significantly regulated and/or significantly methylated. KEGG pathway (Kanehisa et al., 2007) analysis was performed using the human annotation by transferring the gene abundances *via* the unique gene symbols and gene names. We tested for significant overrepresentation of KEGG pathways (Kanehisa et al., 2007) accepting only Bonferroni corrected p values < 0.05.

### Quantitative Real-Time PCR (qPCR)

To validate the gene expression level of selected genes, cDNA was prepared in duplicates using 400 ng total RNA, Improm-IITM reverse transcriptase RNAsin (Promega), and a 3:1 mixture of random hexamers/OligodT. QPCR was performed on cDNA diluted 1:8 on the Biomark HD 96.96 IFC chip (Fluidigm) according to their protocol, and data were collected and processed using the associated software. Raw Ct values were analyzed in GeneEx5 pro (MultiD), and the relative expression levels were normalized to the reference genes; *RPL4* and *TBP*, as these two showed highest stability in the adipocytes according to a Genorm analysis (Vandesompele et al., 2002).

### Immunoblot Analysis

To analyze the relative protein levels in the adipocytes, proteins were extracted from the phenol/ethanol supernatant from the RNA purification according to the manufacturer's protocol (TriReagent) and solubilized in 5 M urea and 0.5% SDS. Protein concentrations were determined using a Pierce BCA assay (ThermoFisher). 20 µg total protein was loaded on a Bis-Tris 10% NuPage gel (ThermoFisher) and transferred to a PVDF membrane using the Invitrogen transfer system (ThermoFisher). Two rabbit polyclonal antibodies against TBC1D16 were used at a dilution of 1:1000. One targeting the N-terminal of the TBC1D16 (aa71–120, ab104407, Abcam) and one targeting approximately the middle of TBC1D16 (aa431–480, LS-C101973, LSBio). Using GAPDH as a loading control and for normalization, the relative protein levels were estimated in ImageQuantTL (GE Healthcare). All three antibodies target human proteins, but they were all predicted to work with the pig TBC1D16 and GAPDH proteins.

### Predicted Transcription Factor Binding Sites

To further decipher the regulatory role of the methylation of six genes (*TBC1D16, PNPLA2, MTOR, PPARA, HLCS*, and *COL6A1*) where the expression level correlated significantly (p < 0.05) with methylation at a specific CpG, an analysis of the predicted transcription factor binding sites were performed in LASAGNA-search 2.0 (Lee and Huang, 2013). Sequences of DNA from 15 bp upstream and 15 bp downstream of the CpG site were inserted into the search tools and analyzed using TRANSFAC TFBSs model v7.0 (Matys et al., 2006), which computes a scoring value for each predicted TF binding sites utilizing a position specific weight matrix mode and a cutoff p value of 0.005.

### Statistical Analysis

Anthropometric and obesity characteristics of the animals were expressed as mean ± SD, and the comparisons between groups were estimated by independent t-tests. QPCR data was log transformed to obtain a normal distribution, and differences in gene expression between groups were also estimated using independent t-tests. Pearson correlation was used for all the correlation presented. Manhattan and QQ plots for the three data sets (mDNAcap, RRBS (5.5), and RNAseq) are enclosed in the **Supplementary Figure S2**. Principal component analysis (PCA) was performed in R using Bioconductor packages **Supplementary Figure S3**. For mDNAcap we used the normalized read counts from diffReps. For RRBS we used only the 2565 methylation sites, which could be called in all samples, and the data were not normalized. The PCA plots for mDNAcap and RRBS are shown in **Supplementary Figure S3**. For comparison sample 584 was removed in both PCA plots.

## Results

### Metabolic Characteristics of Studied Animals

Animal characteristics are shown in **Table 1**. Compared to the lean group, the obese group is characterized by higher body mass index (BMI), abdomen circumference, visceral fat amounts, and circulating total cholesterol and triglycerides. The subset of animals used for the transcriptomic analysis (*RNAseq males*) shows similar anthropometric and metabolic characteristics compared with *all animals* (**Table 1**). Principal component analyses were performed with all the variable characteristics after scaling (**Figure 1**). For all animals, the first two components explain 64% of the variance. Obese males/females are markedly separated from the lean males/females by the first component, which additionally shows a large positive association with visceral fat amount, abdomonal circumference, and weight. The second component shows a negative association with plasma lipids and a positive association with the length and age of the animals. Males used for RNAseq show a similar profile (**Supplementary Figure S3**), where the first two components account for 80% of the variance.

### DNA Methylation Is Altered in Adipocytes From Obese Pigs

To explore if DNA methylation in adipocytes is different between obese and lean pigs, we performed DNA methylation capture (mDNAcap) followed by deep sequencing in mature adipocytes extracted from adipose tissue. On average, we obtained 22 million reads per sample, of which approximately 60% mapped (48% uniquely). We found a total of 7303 differential methylated regions (DMRs) between obese and lean pigs with an adjusted p value < 0.05 (5159 hypo-methylated and 2144 hyper-methylated regions). 30% of these overlap with one or more gene [2207 out of 7303, **Supplementary Table S1(1)**]. To validate our DNA methylation results and further investigate methylation at single base resolution, we performed Reduced Representation Bisulfite Sequencing (RRBS) on the same cell material with a genome wide base coverage of 0.1× to 0.3×. We identified 575 DMRs (p value < 0.05, 182 hypo-methylated and 393 hyper-methylated regions) containing 10 or more CpGs, a mean methylation difference of ≥ 10%, and minimum five pigs in each group (see **Supplementary Table S1(2)**. These regions were assigned to 522 unique nearest genes, with a large fraction (48%) located in gene bodies. Compared to RRBS, our mDNAcap results appeared to be skewed toward hypo-methylated regions, as previously reported (Radford et al., 2014). A less stringent analysis of the RRBS data was performed tolerating only three pigs in each group, but still requiring 10 or more CpGs, and a mean methylation difference of ≥ 10% to call methylation (RRBS-3.3). In this analysis, we identified 2408 DMRs [**Supplementary Table S1(3)**]. We opted to use a more stringent RRBS analysis (RRBS-5.5) in the rest of our analyses.

**Figure 2:**
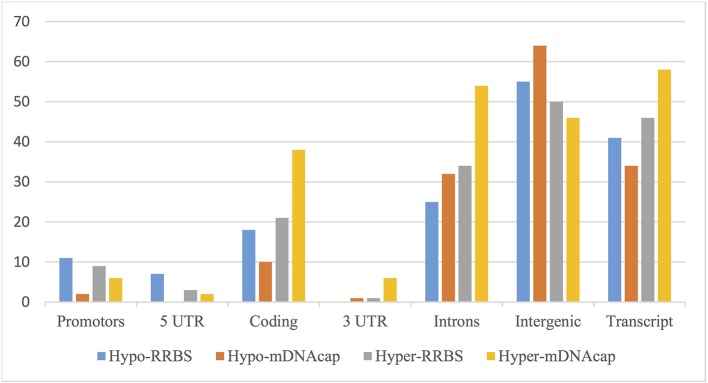
DMRs overlapping different genomic annotations. DMRs from mDNAcap and RRBS are shown as hypo-methylated (decrease methylation in obese animals) or hyper-methylated (increased methylation in obese animals). The overlap percentage is calculated as the number of regions of the given type overlapping an annotation compared to the total number of regions of the given type. As many of the DMRs overlap more than one annotation type, the numbers do not add up to 100% even when the “Gene” annotation is left out.

We next evaluated the distribution of the identified DMRs in both datasets based on their location to the nearest gene. **Figure 2** shows an overview of the genomic distribution of the DMRs with respect to the various genomic annotation types for both mDNAcap and RRBS. Overall, RRBS and mDNAcap data showed similar distribution of the DMRs in respective genome annotations, with approximately 50% of the DMRs overlapping intergenic regions. From the mDNAcap data, obese animals appeared to have less (hypo) methylation in intergenic region, whereas increased methylation was more pronounced within, or at close proximity to genes. The DMRs from the RRBS data show, on the other hand, less methylation in both promoter and in 5′UTR in the obese animals.

Overlapping of the mDNAcap and RRBS data sets resulted in a total of 7985 DMRs of which 3529 are assigned to genes. Interestingly, overlapping DMRs are located at proximity of 173 genes in common between the datasets. However, when comparing the specific location of the DMRs between datasets, only 25 regions overlapped in their positions, and only 17 out of these regions showed parallel methylation between the datasets (15 hypermethylated and 2 hypomethylated regions).

### Gene Expression Is Remodeled in Adipocytes From Obese Pigs

To get insight into the possible influence of the identified DMRs on gene transcription, we performed RNAseq in a subset of 10 animals (5 lean and 5 obese, **Table 1**, **Supplementary Figure S3**). On average, we obtained 100 million reads per sample, of which approximately 80% mapped uniquely. Out of the almost 25,000 annotated porcine transcripts, 1205 were differentially expressed (DE) in adipocytes of lean, compared to obese animals [q < 0.05, **Supplementary Table S1(4)**]. Out of the coding DE transcripts, 708 were downregulated and 497 were upregulated in the obese group.

### Integration of Epigenomic and Transcriptomic Data Sets

**Figure 3:**
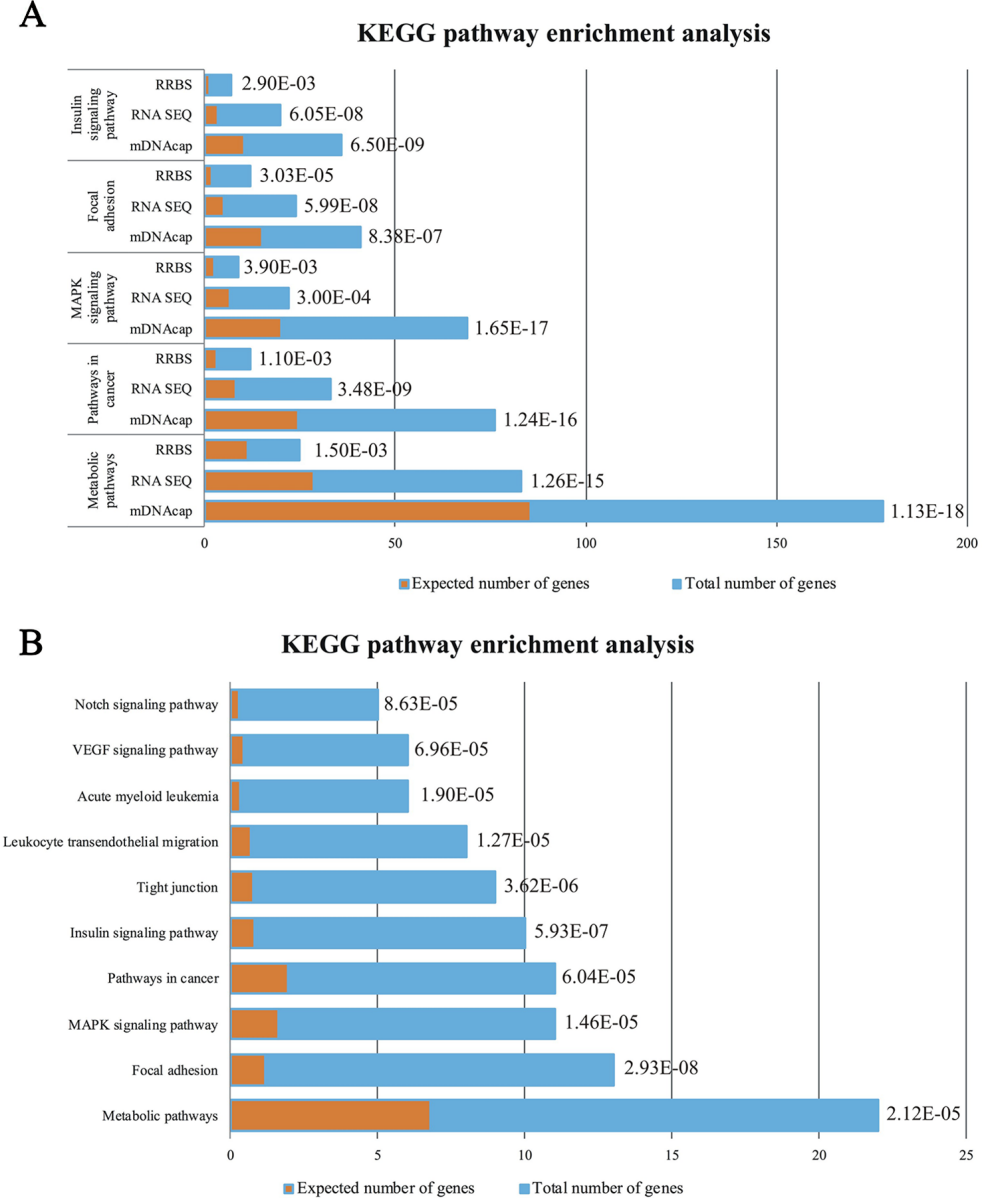
**(A)** Enrichment analysis of the Top5 KEGG pathways in common between the DE genes (RNAseq) and the genes with identified DMRs (RRBS and mDNAcap). **(B)** Pathway analysis showing the significant enrichment of the same five pathways after data was merged, accordingly the DE genes encompassed a least one DMR. Orange columns represent the expected number of genes in the pathways, and blue column display the actual number of identified differential expressed genes/methylated genes in each pathway. The specified *p* values are all Bonferroni corrected.

To get insight into the functional relevance of our data, we performed gene enrichments analysis on all three data-sets (*i.e.* DE genes and genes located nearest to the DMRs). We used official gene symbols of the human genome, since the human annotation information is much more comprehensive than that of the pig. A detailed list of all the over-represented KEGG pathways in the three gene-sets can be found in **Supplementary Table S2(1–3)**. The top five of the enriched pathways genes in common for all three datasets are represented in **Figure 3A**. We identified *pathways in cancer*, *MAPK signaling pathway*, *focal adhesion* as specifically enriched and, most interestingly, other enriched pathways that are highly relevant to the obesity phenotype, such as *metabolic pathways* and *insulin signaling pathway*.

Next, we investigated whether differential expression observed between lean and obese animals is associated with changes in DNA methylation of the nearest DMR. This was done by combining the expression data with the RRBS and mDNAcap data sets. In total, 276 DE genes encompassed a least one DMR, and 29 (11%) of these genes showed differential methylation in both methylation datasets [**Supplementary Table S2(4)**].

Here also, to get insight into a potential biological relevance of these overlapping genes, we performed gene enrichment analysis on the identified 276 DE genes [**Supplementary Table S2(5)**]. Most interestingly, this analysis revealed that KEGG pathways mentioned above (common genes in our epigenomic and transcriptomic analyses), are within the first ten enriched pathways of genes with parallel DNA methylation and gene expression change (**Figure 3B**).

### Validation of Differential Expression

**Figure 4:**
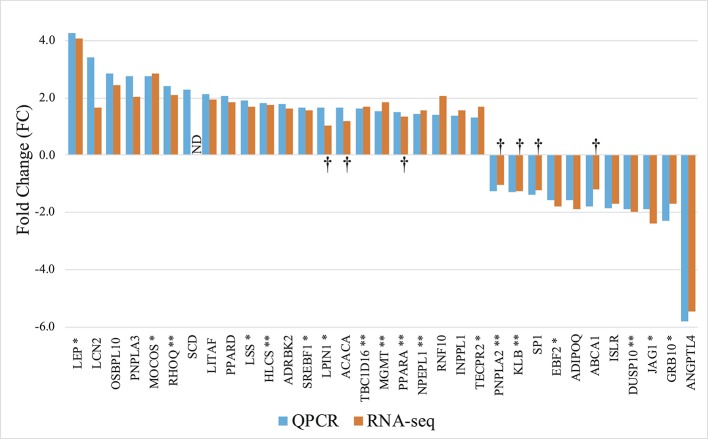
Gene expression profile of the differentially expressed genes in mature adipocytes. RT-qPCR: Orange columns, 9 lean and 10 obese. RNAseq: Blue columns, 5 lean and 5 obese. + FC; Upregulated in obese pigs, − FC; Downregulated in obese pigs. ND, Not determined. †; No significant differentially expression between the groups. *Genes encompassing a DMR. **Genes encompassing a DMR, where the gene expression level correlates with the methylation level (Pearson *p* < 0.05).

To test for the robustness of our findings made in the original cohort, we next investigated expression levels in 9 of the animals from the RNAseq group as well as 10 additional animals using high throughput qPCR. We selected 83 genes based on three criteria: 1) Genes to which statistically significant DMRs mapped (38/83), 2) Genes that were show to be significantly regulated in the RNAseq data (43/83), and 3) Genes implicated in obesity development and immune responses (65/83). The selected genes are listed in **Supplementary Table S3(1)**. RT-qPCR analysis on the 83 genes yielded a total of 33 significantly differentially expressed genes, and all displayed directionally consistent results between the RNAseq and qPCR analyses [**Figure 4** and **Supplementary Table S3(2–5)**]. Of these 33 differential expressed genes, 18 genes encompassed a least one DMR (*p* < 0.05), denoted by ^*^ or ^**^ after the gene name in **Figure 4**.

### Association Between DNA Methylation and Gene Expression

**Figure 5:**
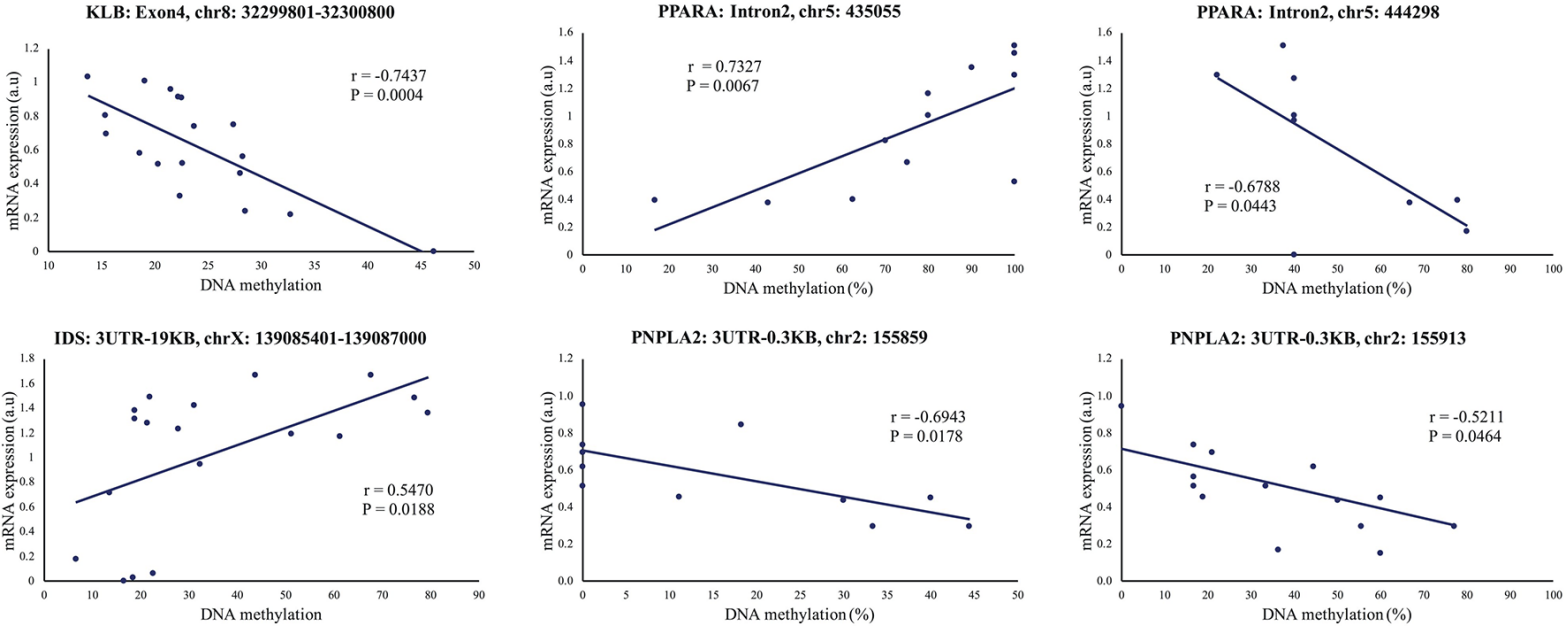
Representative illustrations of significant correlations between gene expression and DNA methylation levels (mDNAcap)/DNA methylation percentage (RRBS) of selected genes. The mDNAcap methylation levels of *KLB* correlate negatively with the gene expression, and gene expression correlates positively with the methylation of *IDS*. In *PPARA* both a positive and a negative correlation is observed between the RRBS methylation percentages and the gene expression level, whereas only negative correlation is seen for the 2 differential methylated CpGs in *PNPLA2*. r is Pearson's correlation coefficient. (See **Supplementary Figures S4**-**S5**) for all the 21 correlation analysis).

To further explore the link between gene expression and DNA methylation level at either specific CpG sites (RRBS data) or the methylation levels of specific regions (mDNAcap data), a Pearson's correlation coefficient (r) was calculated between expression and methylation levels for each of the 18 genes encompassing one or more DMRs (Gene name with ^*^ or ^**^ in **Figure 4**). For 9 of the genes, we found an association between methylation level and gene expression (*r* > ±0.46 and *p* < 0.05). *MTOR*, *SYNE1*, and *IDS*, which almost reached significance level in the gene expression analysis [*p* < 0.1, FC > 1.2, **Supplementary Table S3(2)**], also showed significant correlation between methylation levels and gene expression. Likewise, we found a significant correlation between *COL6A1* expression and methylation. *COL6A1* was significantly downregulated in the RNAseq analysis but not in the RT-qPCR analysis. We opted to include *MTOR*, *SYNE1*, *IDS*, and *COL6A1* in the subsequent analysis. A positive correlation was found for 7 genes: *HLCS, TBC1D16, DUSP10, PPARA, MGMT PNPLA2*, and *IDS*, and a negative correlation was obtained for 6 genes: *RHOQ, HLCS, NPEPL1*, *MTOR*, *SYNE1*, and *COL6A1*. **Table 2** summarizes the correlations between gene expression fold-changes and methylation levels of the DMRs for these 13 genes. Five of these genes exhibit more than one identified DMR correlated with gene expression. Correlation analysis plot was generated for all the 13 genes (21 regions, **Supplementary Figures S4** and **S5**). **Figure 5** shows the correlation analysis between gene expression and DNA methylation for 4 of the genes illustrating the different patterns of methylation-expression correlation. *KLB* shows a strong methylation-induced expression silencing for the mDNAcap DRM in intron 4, whereas methylation of the 3UTR region of *IDS* shows the opposite; i.e. a methylation-dependent increased in gene expression. *PPARA* contains 2 correlated RRBS DMRs, located 9 kb apart in intron 2. The first shows positive correlation, and the second show negative correlation with *PPARA* expression. *PNPLA2* also contains 2 DRMs, located 54 bases apart in the 3UTR, and both showing a negative correlation with gene expression (**Figure 5**). This specify that methylation of CpGs at different location in the gene can have different regulatory functions.

**Table 2:** Differentially expressed genes that correlates significantly with differentially methylated CpG.

Expression					Methylation				Correlation	
Gene	RNA FC	Q-value	QPCR FC	P value	Gene region	ΔDNA methylation	P value	Analysis	Methylation vs Expression	
**RHOQ**	2.10	0.001	2.41	0.000	Intron2	−34%	1.78E−04	mDNAcap	r = −0.57	p = 0.017
**HLCS**	1.75	0.001	1.81	0.000	TSS-30 kb	−51%	1.19E−07	mDNAcap	r = −0.57	p = 0.014
					Intron4	−41%	5.66E−05	mDNAcap	r = −0.50	p = 0.035
					3UTR-3.4 kb	+13%	4.00E−14	RRBS_3.3	r = +0.66	p = 0.039
**TBC1D16**	1.71	0.016	1.64	0.001	Intron3	+12%	7.30E−04	RRBS_5.5	r = +0.55	p = 0.040
					Intron3	+17%	2.10E−13	RRBS_3.3	r = +0.54	P = 0.046
					Intron3	+75%	2.41E−05	mDNAcap	r = +0.48	p = 0.042
					3UTR-22 kb	+ 23%	6.30E−06	RRBS_5.5	r = +0.54	p = 0.025
**NPEPL1**	1.57	0.022	1.45	0.002	3UTR-11 kb	−31%	9.33E−05	mDNAcap	r = −0.57	p = 0.014
**KLB**	−1.25	ns	−1.30	0.003	Exon4	+46%	1.33E−05	mDNAcap	r = −0.74	p = 0.0004
**DUSP10**	−1.99	0.001	−1.88	0.003	TSS-94 kb	−37%	4.90E−05	mDNAcap	r = +0.58	p = 0.013
**PPARA**	1.35	0.184	1.51	0.004	Intron2	+13%	3.00E−06	RRBS_3.3	r = +0.73	p = 0.007
					Intron2	+18%	6.40E−07	RRBS_3.3	r = −0.68	p = 0.044
**MGMT**	1.85	0.001	1.53	0.005	3UTR-29 kb	+45%	3.18E−06	mDNAcap	r = +0.52	p = 0.027
**PNPLA2**	−1.02	0.961	−1.25	0.008	3UTR-0.3 kb	+12%	2.30E−03	RRBS_5.5	r = −0.69	p = 0.018
					3UTR-0.3 kb	+12%	2.30E−03	RRBS_5.5	r = −0.52	p = 0.046
**MTOR**	1.07	0.849	1.17	0.056	Intron26	−29%	1.07E−05	mDNAcap	r = −0.58	p = 0.011
					Intron30	−11%	2.90E−06	RRBS_5.5	r = −0.55	p = 0.050
**SYNE1**	2.36	0.001	1.67	0.089	Intron33	−46%	1.37E−06	mDNAcap	r = -0.51	p = 0.030
**IDS**	1.56	0.023	1.35	0.094	3UTR-19 kb	+47%	1.92E−07	mDNAcap	r = +0.55	p = 0.019
**COL6A1**	−2.50	0.001	−1.72	0.140	Exon1	+13%	6.90E−10	RRBS_5.5	r = −0.65	p = 0.016

ΔDNA methylation; + increase methylation in obesity, − decreased methylation in obesity. Ns, not significant; r = Pearson's correlations. + FC (Fold Change); Upregulated in obesity, − FC; Downregulated in obesity.

**Figure 6:**
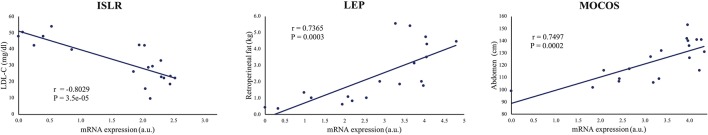
Representative illustrations of significant correlations between gene expression and metabolic characteristic. *ISLR*, *LEP*, and *MOCOS* mRNA levels correlated positively with LDL-cholesterol, retroperitoneal fat and abdomen circumference levels, respectively. r is Pearson's correlation coefficient (See **Supplementary Figures 6**) for the 11 correlation analysis).

For the 24 remaining genes that were significantly regulated between lean and obese animals, for which no correlations were identified, we looked at the potential relationship between changes in gene expression and the changes in metabolic characteristics of the animals, such as the amount of retroperitoneal fat, abdomen circumference, and blood lipid levels (LDL and triglycerides) using Pearson's correlations coefficient. We identified strong and highly significant correlations (r > ±0.6, *p* < 0.005) between the amount of retroperitoneal fat and the expression of *ADIPOQ* (r = −0.65, *p* = 0.003), *GRB10* (r = −0.62, *p* = 0.005), *INPPL1* (r = 0.71, p = 0.0006), *LEP* (r = 0.74, p = 0.0003), *OSBPL10* (r = 0.69, p = 0.001), *RNF10* (r = 0.70, 0.0009), and *SP1* (r = −0.65, *p* = 0.002). Abdominal circumference correlated positively with *MOCOS* (r = 0.75, *p* = 0.0002) and negatively with *EBF2* (r = −0.63, *p* = 0.004). The LDL-cholesterol level correlated positively with *LITAF* (r = 0.74, *p* = 0.0003) and negatively with *ISLR* (r = −0.80, *p* = 3.5e−05). See **Figure 6** for representation of some of these correlations or **Supplementary Figure S6** for all the results.

### Further Analysis of the TBC1D16 Gene

**Figure 7:**
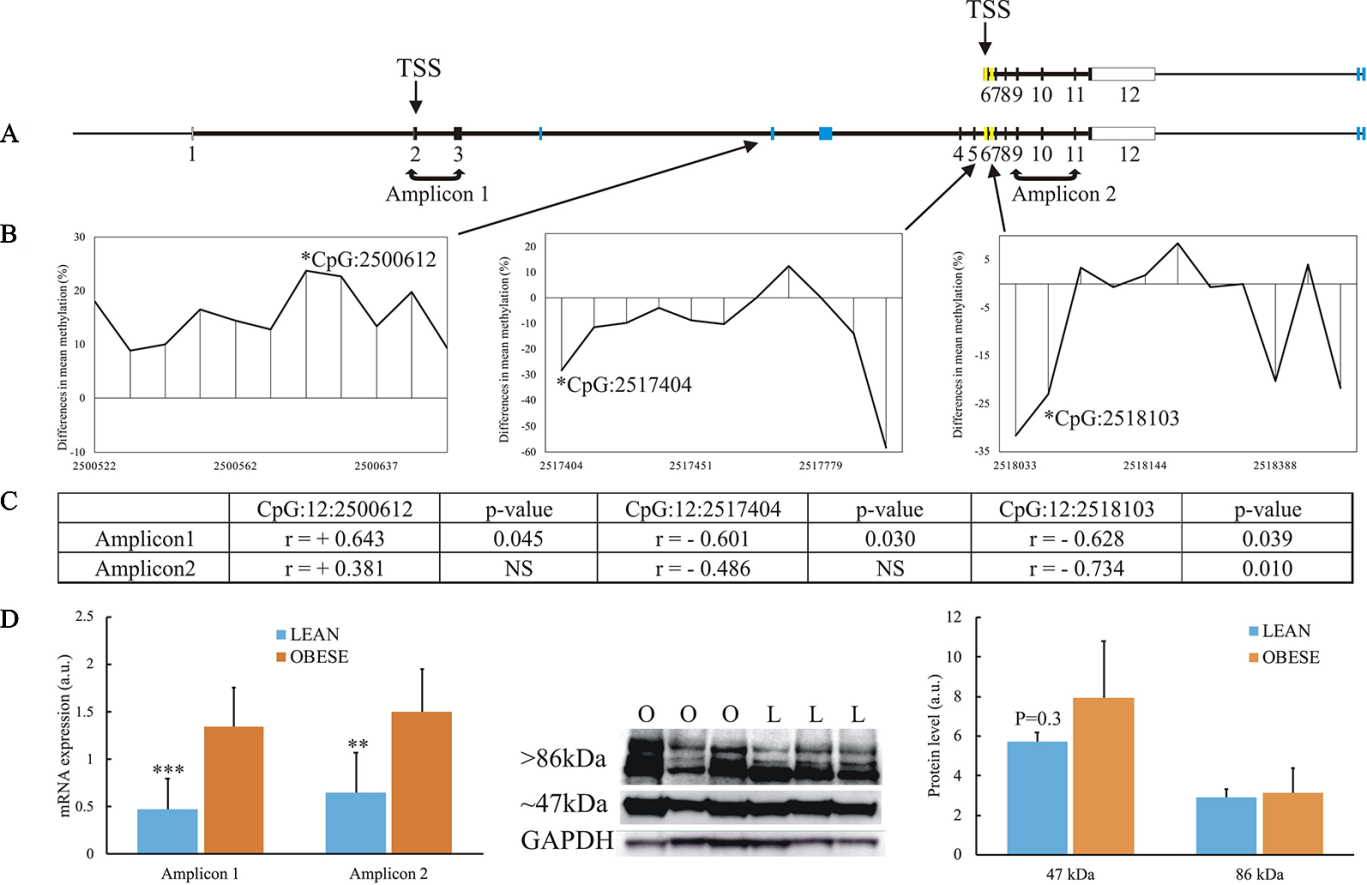
Focus on the *TBC1D16* gene. **(A)** Schematic representation of the small transcript and full-length of the porcine *TBC1D16* gene. Blue boxes represent the location of the 5 DMRs identified in the gene, and yellow boxes represent the additional differentially methylated regions in intron5 and intron6. **(B)** Mean differences in percentage of methylation between the obese and lean animals in 3 DMRs (RRBS), where the methylation levels correlated with *TBC1D16* expression level. Hypermethylation in obese animals are denoted as positive difference, and hypomethylation in obese animals is denoted as negative difference. Each peak represents a methylated CpG. **(C)** The correlation between the methylation of 3 CpGs and expression level of the amplicons targeting only the full-length *TBC1D16* (Amplicon1) and all *TBC1D16* transcripts (Amplicon2), See **Supplementary Figures S7** for the correlation analysis. **(D)** Gene expression and protein expression of porcine TBC1D16. Lean animals; L and blue columns. Obese animals; O and orange columns. NS, Not significant. ** p < 0.0001, *** p < 0.01.

Among the 13 genes showing association between DNA methylation and gene expression, the *TBC1D16* gene was described to be dysregulated in obesity (Pietiläinen et al., 2016; Crujeiras et al., 2017) and contains the highest number of DMRs (5 in total) [**Figures 4** and **7A** and **Supplementary Table S3(4-5)**]. Thus, we aimed to further characterize the epigenetic reprogramming in obesity for this gene. Several isoforms of human TBC1D16 have been identified and annotated, and one smaller isoforms (47 kDa) encoded by an alternative transcription start site (TSS) in intron 5 has been shown to be expressed in human cancer cell lines (Vizoso et al., 2015). The expression of the small isoform is thought to be initiated by hypomethylation in the region around this alternative TSS (Vizoso et al., 2015). To get insight into the possible regulatory role of DNA methylation of TBC1D16 on gene expression, we thus searched at differences in individual CpG methylation in our RRBS data. Interestingly, we identified hypo-methylation in both intron 5 and intron 6 of *TBC1D16* in obese animals (Yellow boxes in **Figure 7A**). Due to poor quality in the annotation of the porcine reference sequence, and little evidence of the short isoform in the RNAseq data, it was not possible to directly infer on an alternative TSS located in intron 5 in the pig. Additional primers were therefore designed to amplify full-lengths transcripts (amplifying Exon2-Exon3, Amplicon 1, **Figure 7A**) in order to compare the level of this transcript to the level of all transcripts target by Amplicon2 (amplifying Exon9 to Exon11, **Figure 7A**) to determine whether the small isoform is present at a higher quantity in the obese state. Both amplicons were significantly upregulated in obese animals, with equivalent fold-changes (**Figure 7D**). However, when calculating the correlation between expression of Amplicon1 or Amplicon2 and methylation level of the three CpGs in intron3, intron5, and intron6, we found stronger correlation with Amplicon1 compared to Amplicon2, as all 3 CpGs were correlated with Amplicon1 expression and only one CpG (in intron6), was significant correlated Amplicon2 (**Figures 7B, C** and **Supplementary Figure S7**). Next, we determined TBC1D16 protein levels in adipocytes of obese and lean animals by Western blotting. A ∼ 86 kDa protein (corresponding to the expression of the large transcripts of *TBC1D16*), was detected at similar levels in three lean and three obese animals. Conversely, a protein of ∼ 47 kDa (corresponding to the short transcript), displayed a trend to be higher in adipocytes from obese animals, (p = 0.24) which agrees with the differential expression obtained from both RNAseq and RT-qPCR results (**Figure 7D**).

### Transcription Binding Site Analyses

**Figure 8:**
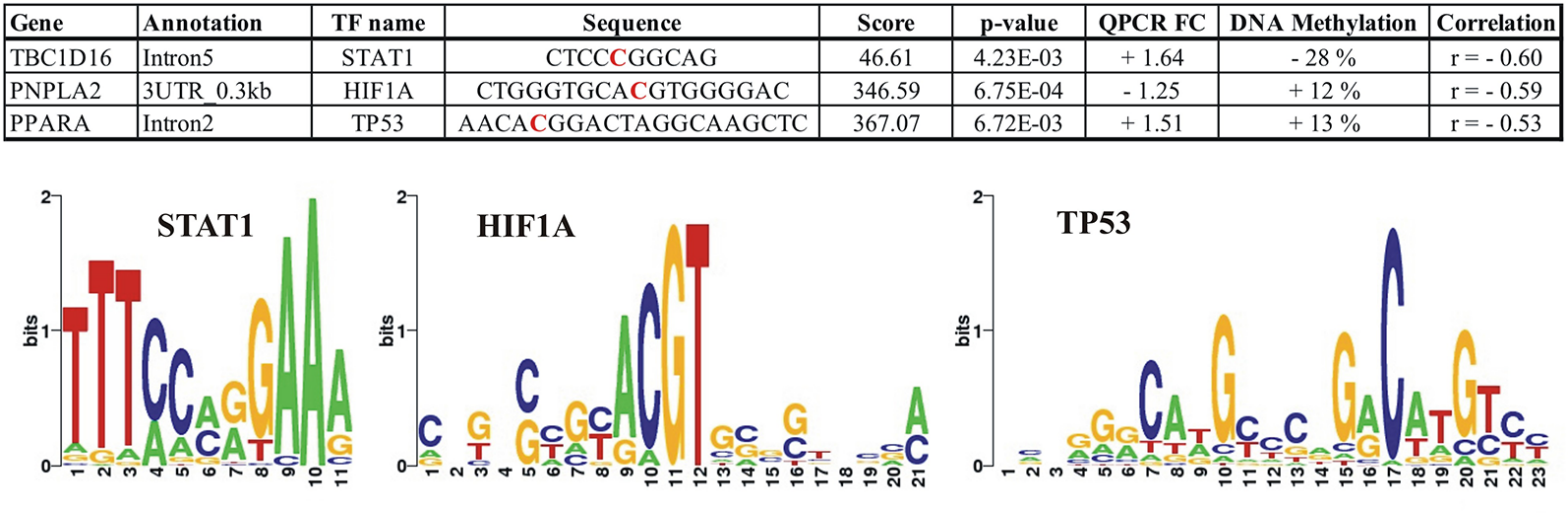
Illustration of the predicted putative binding sites for the transcriptions factors; STAT1, HIF1A, and TP53 within the differential methylated CpGs located in the *TBC1D16*, *PNPLA2*, and *PPARA* genes, respectively. The red bases in the sequences correspond to the positions where differential methylation is identified. For *TBC1D16*, this position corresponds to position 6 in the STAT1 motif, and for *PNPLA2* the differential methylated CpG is localized in position 10 in the HIF1A motif. The differential methylated CpG in PNPLA2 is localized in position 8 in the TP53 motif. + FC (Fold Change); Upregulated in obesity, − FC; Downregulated in obesity. DNA methylation; + increase methylation in obesity, − decreased methylation in obesity.

To identify transcription factor (TF) networks that could influence the expression of the identified genes, we used the tool LASAGNA (Lee and Huang, 2013) to search for putative TF binding sites on DNA regions showing a significant correlation between the methylation level at a specific CpG (RRBS) and expression of the nearest gene. We identified putative binding sites for TFs in all of the identified correlated RRBS CpGs and also in intron5 of *TBC1D16*, where approximately half of the identified methylated CpGs contained putative binding sites for multiple TFs. We established the predicted TFs presence in the adipocytes (RNAseq data) and ranked the TFs according to their p-value calculated by the TRANSFAC TFBSs model. Data for all predictions are presented in **Supplementary Table S4**. **Figure 8** illustrates how *TBC1D16*, *PNPLA2*, and *PPARA* contain consensus-binding sites for the 3 TFs; STAT1, HIF1A, and TP53, respectively.

## Discussion

We established the genome wide DNA methylation profile of adipocytes from lean and obese pigs using two different technical approaches and conducted a comprehensive integrated analysis of the epigenomic data with the transcriptome from the same adipocytes. We found that methylation pattern of adipocytes of obese pigs is altered in 7985 regions, that we assigned to 3529 unique genes. The genomic distribution of the identified DMRs is consistent with the results reported in similar studies, that is, fewer near promoter regions and 5′- and 3′UTRs, and the majority in exons, introns, and intergenic regions (Arner et al., 2015).

Here we identified genes already described as playing a role in obesity but also, we discovered several new candidate genes that may be involved in the development of obesity. Interestingly, the most significant DMR (*p* = 3.7E–14) in our RRBS data analysis between obese and lean animals, is localized in exon1 of the *SOCS4* gene [**Supplementary Table S1(2)**], where methylation is increased by 13% in obese animals. The RNAseq data revealed that this gene is also significantly upregulated in obese animals [**Supplementary Table S1(4)**]. There are eight mammalian SOCS proteins; SOCS1–3 has been linked to regulation of cytokine signaling, whereas SOCS4–7 has been associated with grow factor receptor signaling (Trengove and Ward, 2013). Since we did not validate and confirm the *SOCS4* expression by qPCR in additional animals, only a subset of the animals shows that the *SOCS4* expression in adipocytes is reprogrammed by DNA methylation changes.

Four of the most significant mDNAcap regions are located in the *SNORD116* region, where hypermethylation is seen in the obese animals [**Supplementary Table S1(1)**]. Our RNAseq data also revealed a marked upregulation of the *SNORD116* transcripts in obese animals (FC = 2.2, padj = 2.89E–4, however the expression is relatively low in the adipocytes [BaseMean = 10.4, **Supplementary Table S1(5)**]. The *SNORD116* cluster is part of an imprinted chromosomal segment, that is critical to the development of Prader Willi syndrome (PWS) (Fontana et al., 2017). PWS is a disorder that causes, among other dysfunctions, life-threatening obesity. Since *SNORD116* is mainly expressed in the brain, where it is involved in the regulation of food intake (Qi et al., 2016), it is unclear whether this epigenetic remodeling of SNORD116 in the adipocytes is of great importance. Several studies have compared methylation patterns in across tissues and cell types, and found that methylation consistent profiles, notably between circulating leukocytes and others tissues (Bacos et al., 2016; Crujeiras et al., 2017; Wahl et al., 2017). Whether *SNORD116* is also hypermethylated and upregulated in the brain of the obese pigs remain to be investigated.

Functional enrichment analysis of DMRs and DE genes shows that pathways altered in adiposity are similar to what is described in matching human obesity studies. Genes related to metabolic, cancer, focal adhesion, MAPK- and insulin signaling are among the most significant over-represented pathways (Arner et al., 2015; Benton et al., 2015; Dahlman et al., 2015; Ronn et al., 2015). These findings are also in accordance with a study comparing the differences in adipose tissue methylation between a lean pig breed and an obesity prone pig breed, in which the same pathways have been implicated (Yang et al., 2016). Of interest, in the present study, pathways associated with inflammation and the immune system were not among the most significantly over-represented pathways in the functional enrichment analysis. This is likely due to the fact that we conducted our investigation in isolated mature adipocytes, whereas most studies investigated whole adipose tissue, where infiltrated immune cells probably have a large impact on the overall methylation profile (Nilsson et al., 2014; Ronn et al., 2015; Pietiläinen et al., 2016).

We validated 58% (25/43) of the DE genes by high-throughput RT-qPCR. Several of these genes, like *LEP*, *PPARA*, *PPARD*, *LPIN1*, *SREBP1*, and *ADIPOQ* are described in obesity in both humans and mice. To our knowledge, the *MGMT*, and *NPEPL1* genes, for which the gene expression levels correlated significantly with methylation level, have not been associated with obesity. Not much is known about *NPEPL1* (aminopeptidase like 1), a gene genomically localized between *STX16* and *GNAS*. *GNAS* is an imprinted locus, and both imprinting inaccuracies in the *GNAS* locus and chromosomal deletion of the *STX16* gene give rise to the same disease phenotypes, notably increased adiposity (Grüters-Kieslich et al., 2017). This observation makes *NPEPL1* a potential novel candidate gene of interest for obesity physiopathology. In addition, *MGMT* (O-6-methylguanine-DNA methyltransferase), is known to participate in the defense against mutagenesis and toxicity from alkylating agents. Methylation of the *MGMT* promoter has been linked to the development of several forms of cancer (Sharma et al., 2009). We found that *MGMT* is upregulated in obese animals, and we detected a correlation between *MGMT* expression and methylation levels, which suggest that methylation of *MGMT*-DMR regulates *MGMT* expression. Given the important increase in oxidative DNA damage in obesity, upregulation of *MGMT* may be involved in the repair of DNA damages caused by obesity.

*TBC1D16* is also highly associated with the development of cancer due to hypomethylation in intron 5 of the gene that has been shown to reactivate a small transcript of *TBC1D16* (TBC1D16-47KD), which exacerbates melanoma growth and metastasis (Vizoso et al., 2015). Interestingly, we also see an upregulation of the *TBC1D16* transcripts and hypomethylation in the same location as in humans. However, due to both insufficient coverage of reads and incomplete assembly of the porcine reference genome in this region, we were not able to amplify the small transcript (TBC1D16-47KD), which may be regulated by methylation. Nevertheless, the protein expression of TBC1D16-47KB indicates a higher level of this isoform in the obese animals. *TBC1D16* is also found significantly differentially methylated and regulated in obesity in two others studies (Pietiläinen et al., 2016; Crujeiras et al., 2017), but neither of these studies investigated the expression level of the small transcript (TBC1D16-47KD). Given that TBC1D16 is involved in the canonical PI3K/AKT pathway, which is found deregulated in both obesity and cancer, the activation of TBC1D16-47KD might be one of the links between obesity and cancer.

Here, we found that 13 genes show a correlation between methylation and gene expression level, of which 10 genes showed inverse correlations (i.e. increased methylation associated with decreased expression, or decreased methylation associated with increased expression, **Table 2**). Only *TBC1D16*, *DUSP10*, *IDS*, and *PPARA* showed positive correlations. The common appreciation is that methylation in promoter regions represses transcription of the nearby gene, and that methylation in gene bodies contributes to higher transcription (Jones, 2012; Yao et al., 2015; Grunert et al., 2016). The correlations we established provide further evidence that methylation can both stimulate and repress transcription, depending on the genomic localization of the epigenetic change. This is for example apparent in *PPARA* intron2, where both a positive and a negative correlation is shown, and these two differential methylated CpGs are only 9 kb apart (**Figure 5**). For *MGMT*, methylation was found in the gene body (intron2), which is in line with the higher gene expression levels detected for that gene.

By using LASAGNA to identify potential transcriptional regulators, we found putative binding sites for TFs in all six genes inspected. **Figure 8** shows three of the potential TFs (STAT1, HIF1A, and TP53) that may regulate expression of the identified genes. HIF1A and TP53 are both well-known to regulate several genes involved in glucose and fatty acid metabolism, and are both expressed in adipocytes. Of note, binding of both HIF1A and TP53 have been shown to be methylation-sensitive TFs (Domcke et al., 2015). Expression of *PNPLA2* correlates negatively with the methylation of a CpG site located within the 3UTR region, which perfectly match with the knowledge that HIF1A is mainly activator of transcription. The differential methylated CpG in the binding site is localized in position 10 in the HIF1A motif (**Figure 8**), which is one of the most conserved bases in this motif. Thus, increased methylation in this CpG position could block HIF1A binding, and thereby repress the transcription of *PNPLA2*.

We found STAT1 is predicted to bind directly in the differential methylated CpG in intron 5 of *TBC1D16*. STAT1 is regarded as a transcription activator (Satoh and Tabunoki, 2013) binding of STAT1in promoter region and 5UTRs of *TBC1D16* in obesity could therefore regulate the TBC1D16-47KB transcript as described earlier; hypomethylation of the STAT1 binding site could increase the binding of STAT1 and thereby, activate transcription of the short isoform of *TBC1D16*. Of potential interest, comparative mapping of the pig and the human genome indicates that *TBC1D16-DMR* in pig corresponds to a region in the human genome (17:79950940-79950840CRGh38.12), where both a promoter (ENSR00000565975) and a CTCF binding site (ENSR00000565978) are annotated. These results support that differential methylation in this region in the *TBC1D16* gene could have a direct impact on the expression of the small TBC1D16 transcript.

## Conclusion

In this study, we have extensively characterized DNA methylation in a cell-type specific manner. We show that mature adipocytes differ between lean and obese individuals. Our data support that altered methylation in obesity may play a role in the perturbation of specific pathways involved in obesity and obesity-related metabolic diseases. The association between methylation and expression of genes implicated in obesity and obesity-related metabolic diseases supports a role of epigenetic remodeling in the development of obesity. Our cell-type specific analysis, using an animal organism with strong common physiological characteristics to humans constitutes a valuable resource for future work investigating further the role of DNA methylation in the etiology of obesity and associated disorders.

## Data Availability Statement

The datasets generated for this study can be found in the Gene Expression Omnibus (GEO) database GSE136754.

## Ethics Statement

All research involving both animal and tissue sampling were conducted according to the Danish “Animal Maintenance Act” (Act 432 dated 09/06/2004) and with the approval from the Danish Animal Experimental Board (J.nr 2007/561-1434).

## Author Contributions

MJ, RB, MF and CJ conceived and designed the study. MJ, SC, PK-M, CB, CM and MF accomplished the sample collection and MJ, CM, PK, PL, MG and SC performed the experiments. MJ, JH, CA, CM, SC, SP, CJ, RB and JG contributed to the analysis and interpretation of data. MJ wrote the manuscript. All authors revised critically and approved the manuscript.

## Funding

This study was supported by The Danish Independent Research Council (FTP 0602-01742B and DFF 1335-00127), The Lundbeck foundation (R34-A3587), Innovation Fund Denmark (0603-00320B). The Novo Nordisk Foundation Center for Basic Metabolic Research (http://www.cbmr.ku.dk) is an independent research Center at the University of Copenhagen, partially funded by an unrestricted donation from the Novo Nordisk Foundation.

## Conflict of Interest

The authors declare that the research was conducted in the absence of any commercial or financial relationships that could be construed as a potential conflict of interest.

## Supplementary Material

Supplementary Table S1 mDNAcap, RRBS, and RNAseq.

Supplementary Table S2 Gene Enrichment Analysis.

Supplementary Table S3 QPCR validation data.

Supplementary Table S4 TF binding predictions.
