# Supplementary material for: Epigenetic and Transcriptomic Characterization of Pure Adipocyte Fractions From Obese Pigs Identifies Candidate Pathways Controlling Metabolism
Source: Front Genet. 2019 Dec 17;10:1268. doi: 10.3389/fgene.2019.01268 (PMC6927937; doi:10.3389/fgene.2019.01268)

Supplementary figures:

S1:

A representative microscope picture of the isolated mature adipocytes.

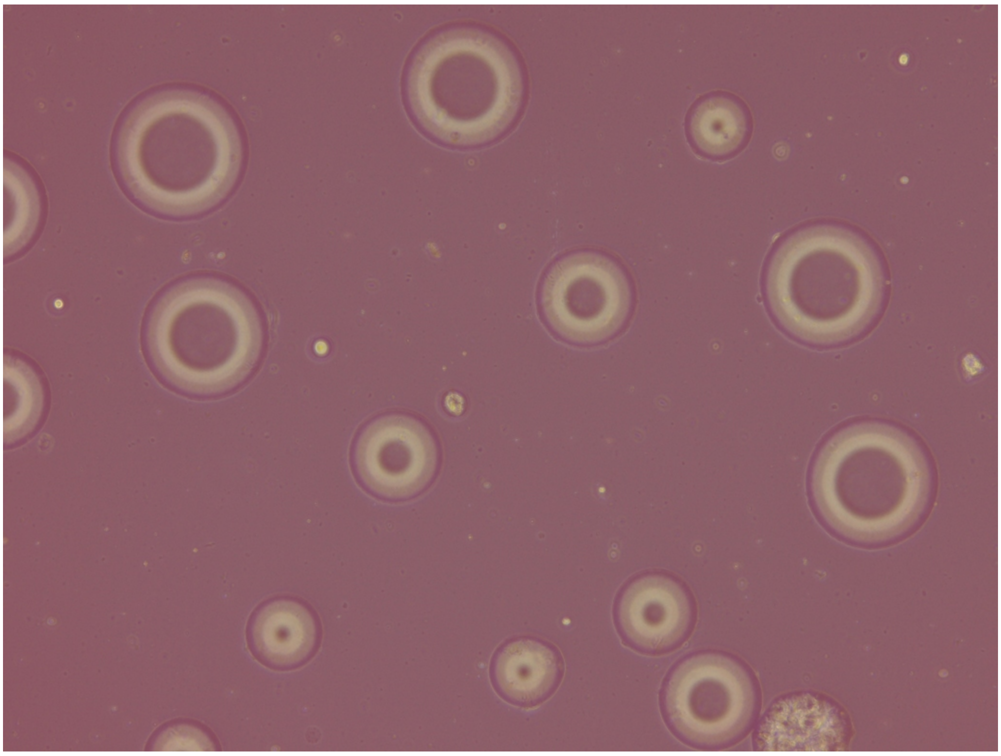

S2:

Manhattan plots of mDNAcap, RRBS and RNAseq data:
The figures show -log P-values and -log Q-values plottet agains the chromosomal positions.

mDNAcap:

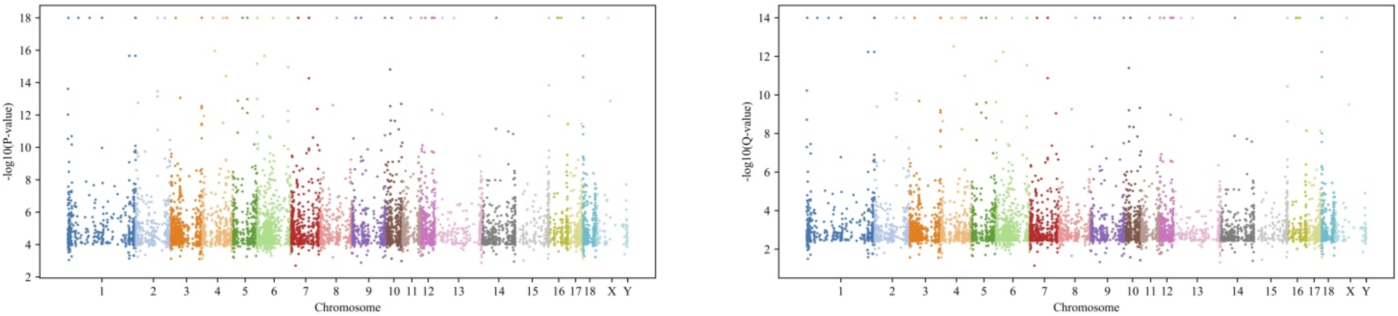

RRBS_default:

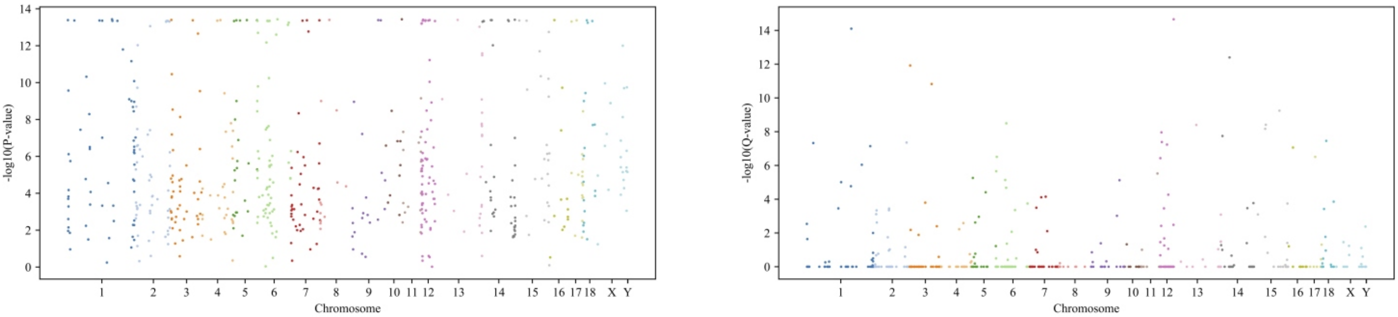

RNAseq:

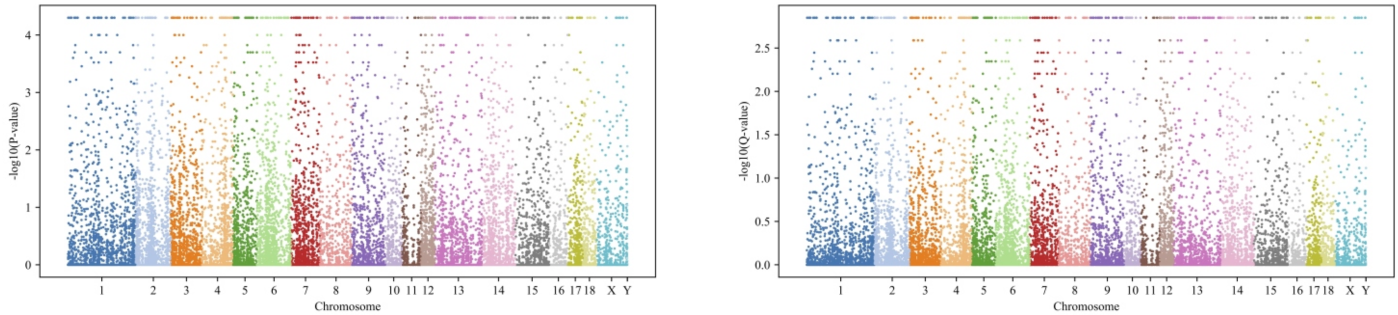

QQ plots of mDNAcap, RRBS and RNAseq data:
The figures show the observed -log10 of the p- and q-values versus -log10 of the expected p- and q-values. (The expected values were calculated as (2*rank -1)/2*n, where rank is the observed value after sorting by the value).

mDNAcap:

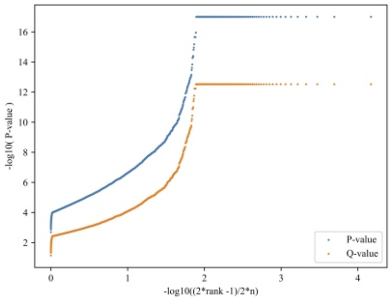

RRBS_default:

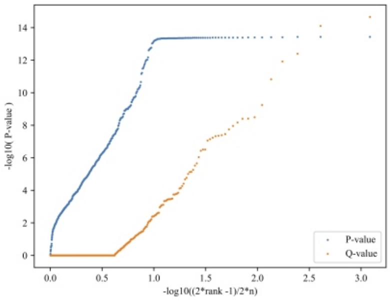

RNAseq:

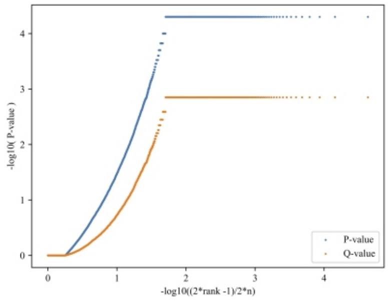

S3:

Biplots of the PCA of the anthropometric and metabolic characteristic of the RNAseq males used.

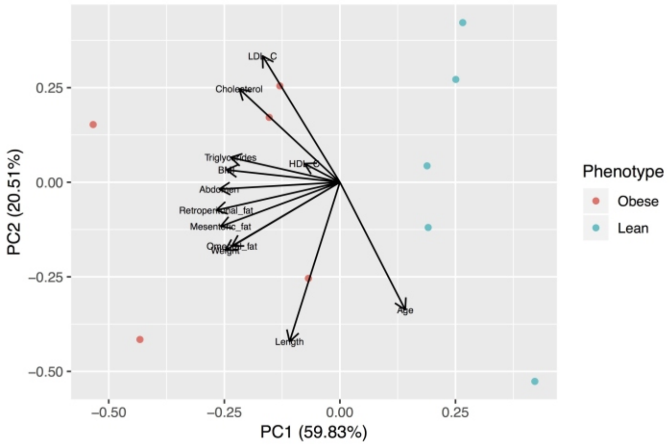

PCA plots of mDNAcap, RRBS and RNAseq data:

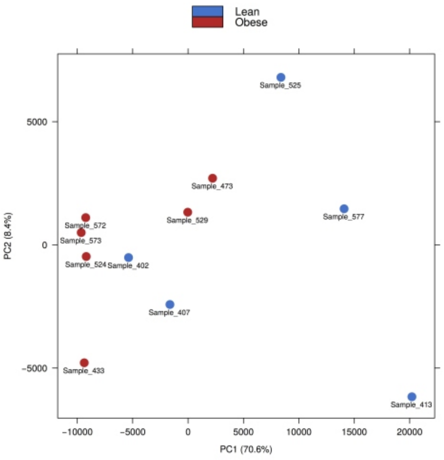

PCA plot of RNAseq

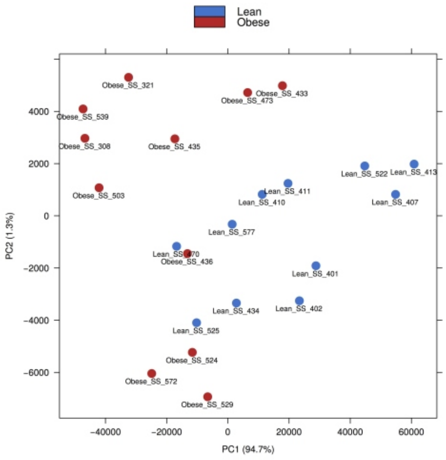

PCA plot of mDNAcap

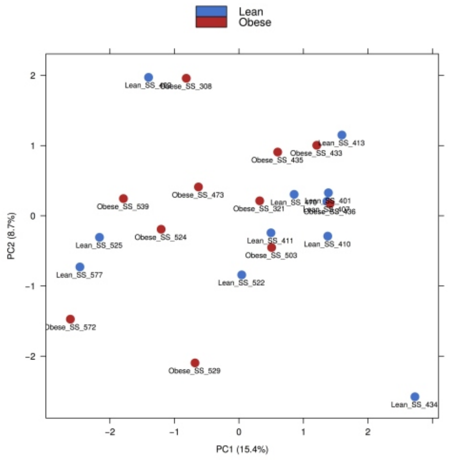

PCA plot of RRBS

S4:

Correlation analysis between the differential expressed genes and differential methylated CpGs:

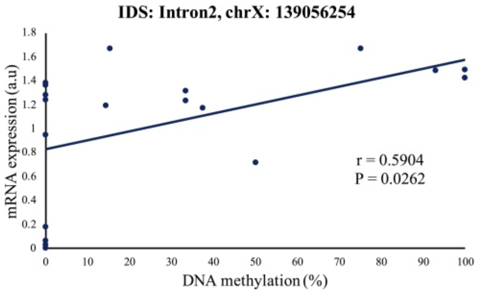

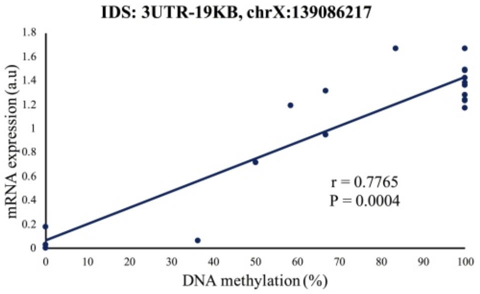

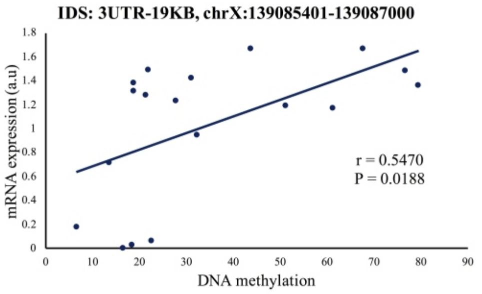

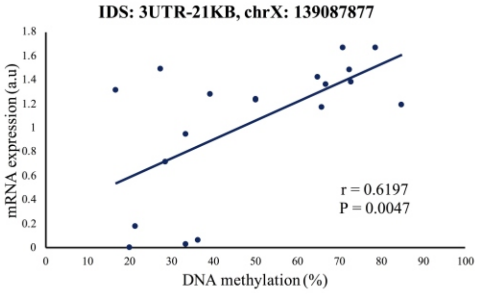

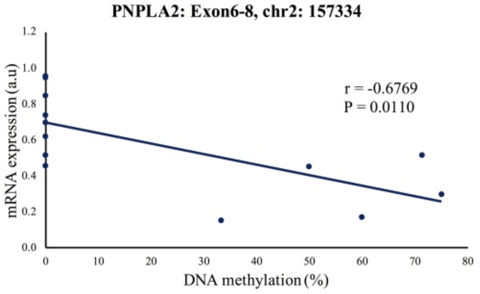

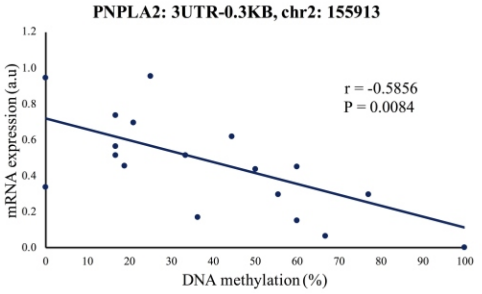

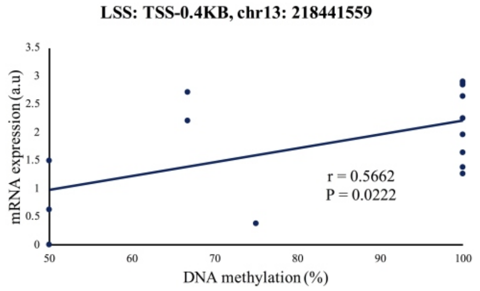

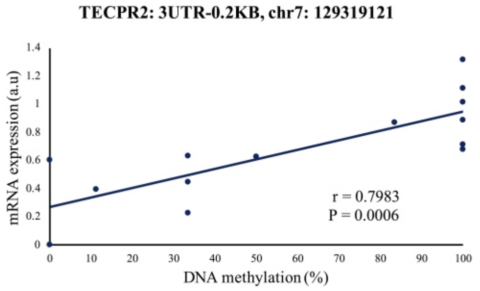

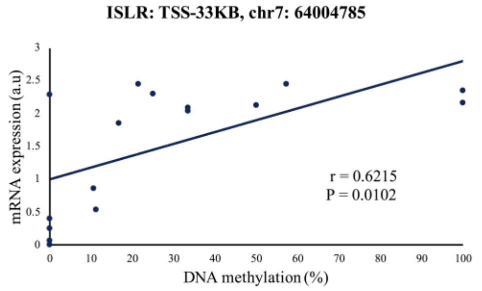

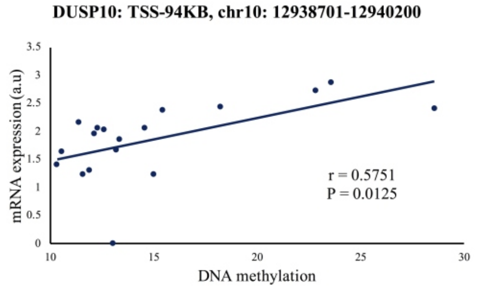

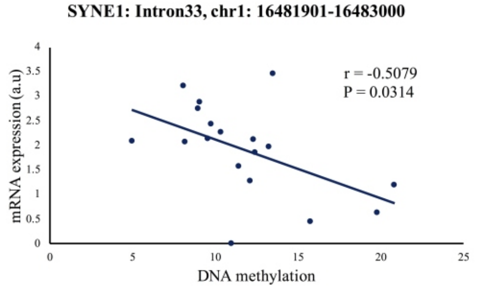

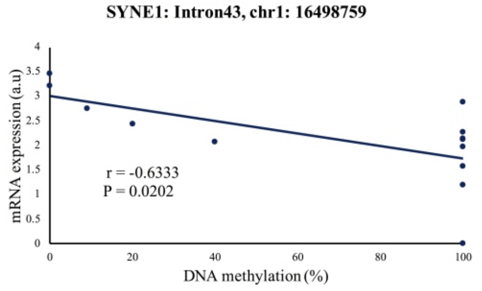

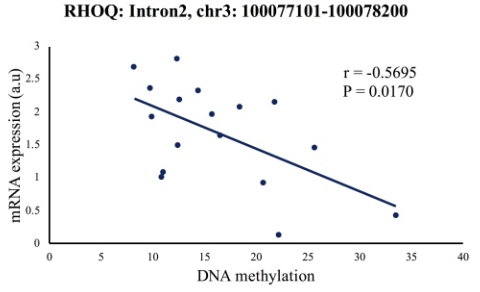

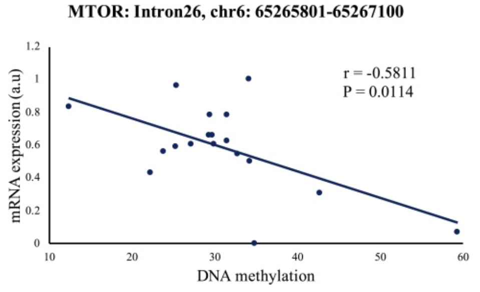

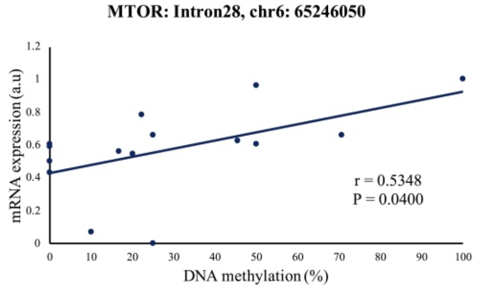

S5:

Correlation analysis between the differential expressed genes and differential methylated CpGs:

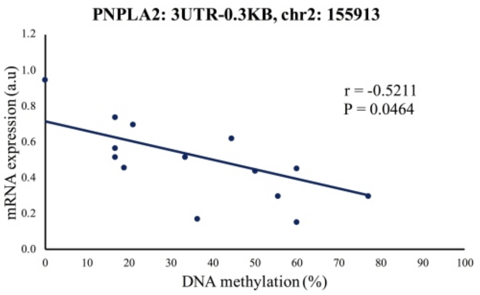

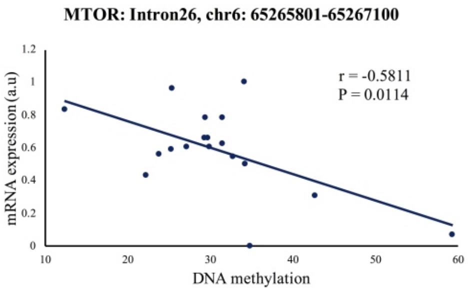

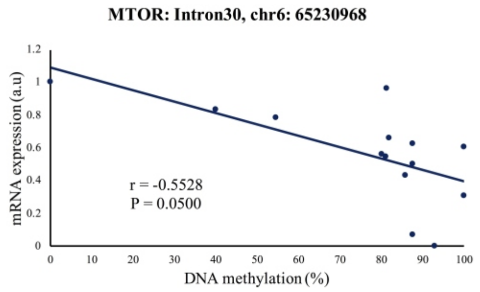

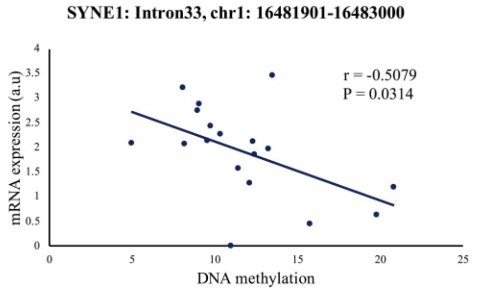

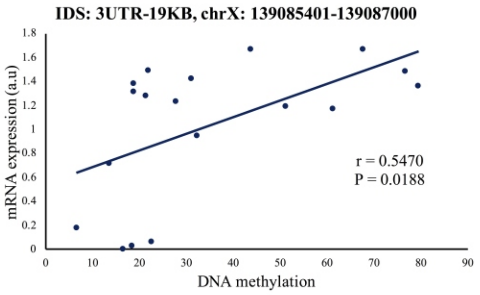

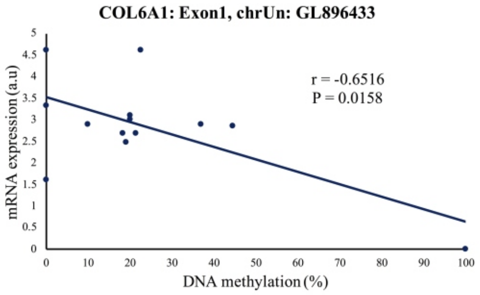

S6:

Correlation analysis between the differential expressed genes and the metabolic characteristic:

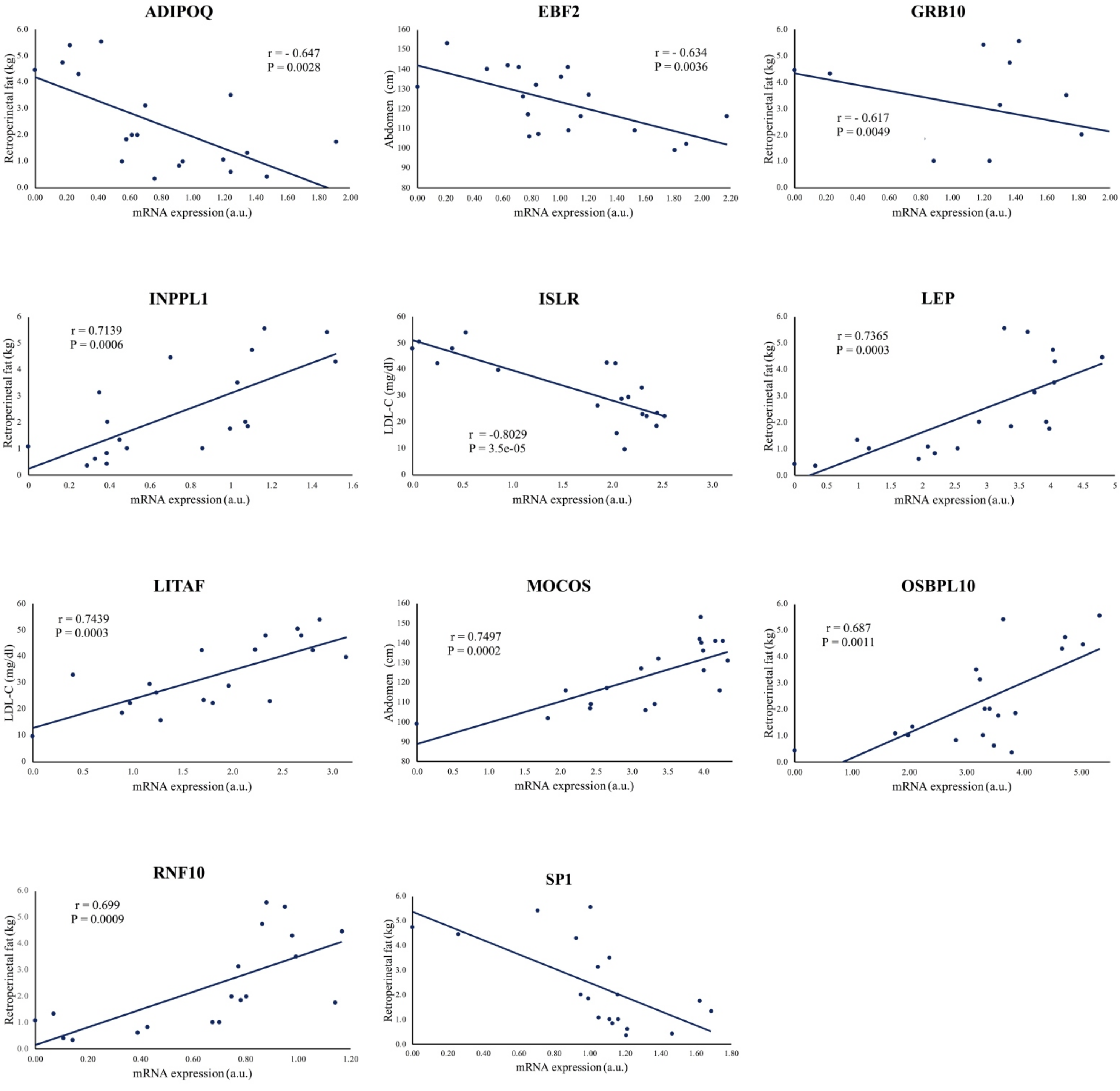

S7:

Correlation analysis between TBC1D16 Amplicon1 and Amplicon2 and the differential methylated CpGs:

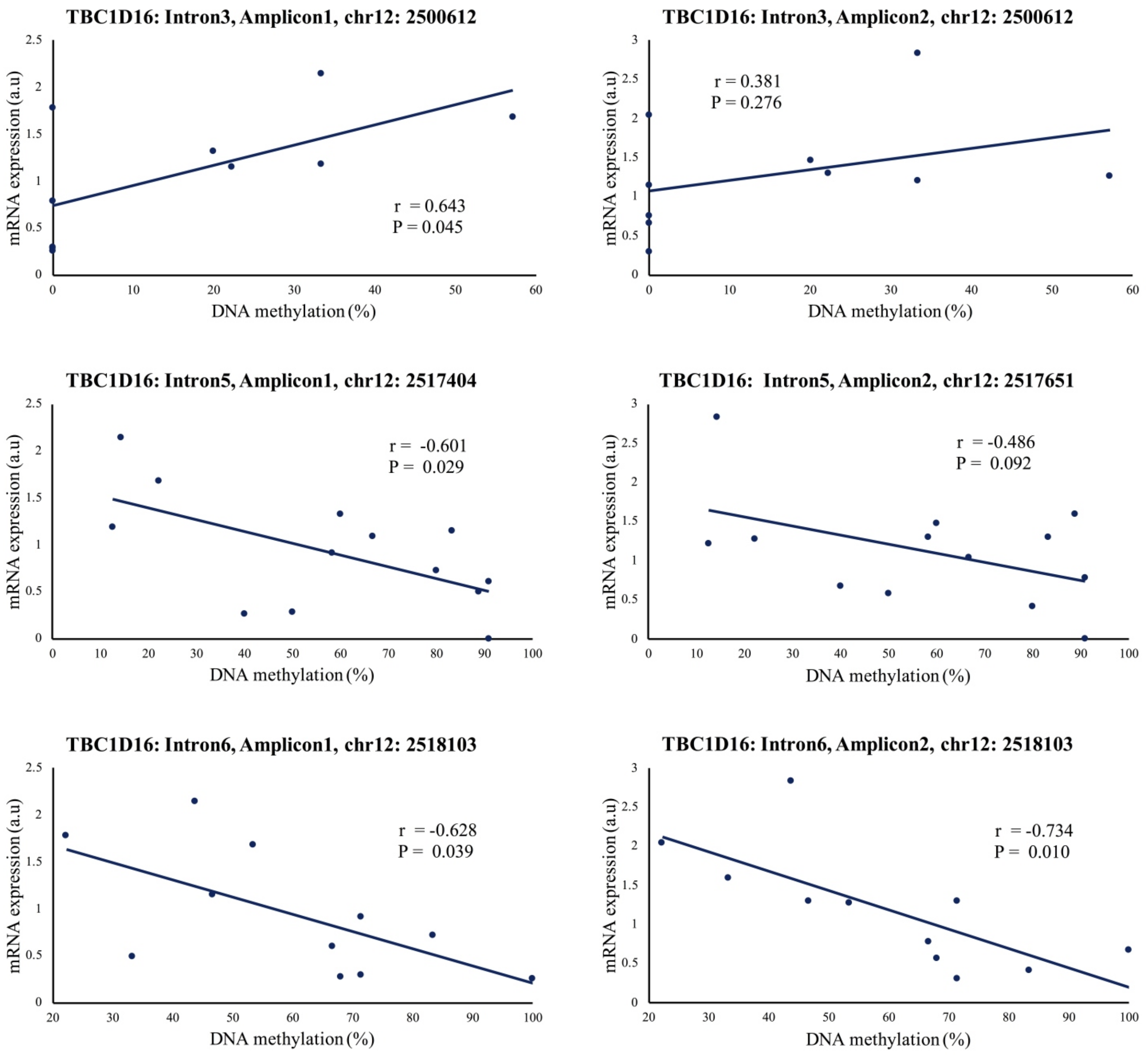

Supplement: Supplementary file 1 [file DataSheet_1.pdf]
